# Is There Too Much Surgery?

**Published:** 1910-07

**Authors:** W. W. Mangum

**Affiliations:** Rome, Ga.


### IS THERE TOO MUCH SURGERY?

BY DR. W. W. MANGUM, A. M., M. D., ROME, GA.

It gives me great pleasure to appear before you to-day, not as a visitor as in the past, but as a member of the Georgia Association. During the years of my residence in Alabama, I learned to know some of you intimately; with others of you, I have from time to time, during the past ten or twelve years, had convention acquaintance; appearing on the programme of different medical organizations, and enjoying an occasional tilt over some disputed method of procedure. It might have been better for me to have chosen a subject on this, my first, appearance before you as a member of this body, which would bring about a more harmonious discussion; but I have for a number of years had in mind the purpose to unburden myself of my views on this particular question, and so I am here with no apologies to make.

Is there too much surgery? Well, now, that is a debatable question, isn't it? In taking the affirmative side in this discussion, I trust that I am not creating the impression in your minds that I am not appreciative of the great perfection of method attained by this branch of our profession; nor of the great saving of life resulting from the pioneer work of certain eminent men. Quite the contrary. I feel always like uncovering my head when the name of McDowell or Sims is mentioned in my hearing. These were great men, and we have had many like them. I speak of these two only because they were from our own section. If I were not limited in my time allowance, I would indulge myself in paying tribute to modern surgery; and I would attempt to show how "the damned butchery," of one hundred years ago, has been changed to the marvel of the twentieth century. However, this is not my subject; and it is only necessary to say that I am thoroughly cognizant of the fact that it is the exact branch of medicine.

In this argument, I shall first consider the fact that a great many cases come to the surgeon which should not have been allowed to become surgical. This statement may seem rather vague until you get at the point I am trying to make. At different times during the past few years, I have questioned prominent surgeons in the South and East as to the percentage of strictly gynecological cases in the patients brought to them for relief, and from all of them I have had the unvarying answer that the proportion was very large. A second question that I usually ask is: "What percentage of this class of work could have been prevented by careful and intelligent attention on the part of the family physician?" The usual reply was "Ah, now, Doctor, you would not have me criticise my friends."

For eighteen years I have been a general practitioner. I have never tried to be anything else. And during those years I have had some queer experiences with men who hold a State license to practice. I have been called in consultation to patients in country districts, where the doctor in attendance was treating a case of prolapsed womb with porous plasters on the back. I have seen cases of dysmenorrhoea, due to a retro-flexed uterus, treated with some of the St. Louis proprietary cure-alls. I have seen a pruritus, due to a gonorrhoeal infection, treated with a dusting powder. I have seen cases of chronic ovaritis being indulged with the comforting thought that it would be all right after the change of life. I have seen women allowed to leave their beds a week after confinement, and resume their household duties. I have seen many things that have made me oftimes want to say to the attending physician: "Please tell me what college graduated you?"

I am persuaded that I am not alone in this experience, and I am merely stating facts, not attempting to suggest relief. That must be attended to by our college faculties and State Examining Boards. Such patients as these necessarily fall into the hands of the surgeon for plastic and reparative work, for Alexander operations, for ovariotomies and removal of pus tubes, etc., etc., when a little skilled attention at the proper time would have restored the woman to health and strength. It is from such sources as this that the high percentage of gynecological work comes. This is one of the conditions on which I base the argument that there is too much surgery, too much that should not have become necessary, and on this division of my subject I am sure there is no difference of opinion. There is much that can be said along this line, but it is an unpleasant task, and I shall not enlarge on it, more especially as I am assured in advance that on this we are all agreed.

Concerning the other division of my subject, I am not so sure of a unanimity of opinion. I suspect that it is an unpopular position that I shall take. Nevertheless, it is an opinion formed after years of close study of conditions, and as we are met here for a discussion of the needs of our patients, I am assured, at least of a respectful hearing.

The second reason why I am of the opinion that there is too much surgery, lies in the fact of the tendency toward an unconscious bias that exists in many men who make a special study of the use of the knife. It takes a great man to be a specialist. Conservatism should be the badge of the specialist, not of the general practitioner. And it takes a great man to be conservative. The small man in politics or in war becomes restless in the face of danger, and suggests a radical move; but the finished statesman and the experienced soldier know that "discretion is the better part of valor," and that oftimes a waiting game brings victory when a sudden move might precipitate disaster.

The discussion of the deplorable results occasioned by the ignorance or indifference of some general practitioners, was intentionally put first in this paper, for the reason that I wish to show that the unfailing supply of material furnished by this sort of men, has suggested to some of their better equipped brethren in every community to take on surgery as a side line, so to speak; and while attending to the ordinary ills of their patients, to furnish them with the opportunity of disposing of their ovaries and appendices, should the occasion require. These men, many of them, do good work, with certain limitations; and afford relief to a class of people in many instances, who are not financially able to go great distances, and employ great surgeons. I repeat, these men do good work, and fill a long felt want, but there is a great danger they have to avoid; and this brings me to the discussion of what manner of men should do our surgery.

We are all brothers here, and brothers can say things to each other which they would resent if coming from an outsider; so I shall speak plainly. Years ago, when this private hospital business began really to attract the attention of the people, we general practitioners would call in our brethren who conducted an institution of this character, whenever we had a patient with some obscure condition which failed to respond to ordinary remedies. These brethren would find some internal—usually some pelvic organ responsible for the entire trouble, and advise its immediate removal. We general practitioners didn't know as much then as we do now, and so the organ would be removed. It was all very nice and plain, while it was being done, and we family physicians would get the anaesthesia fee, the rest of the bank account usually going to the operator. But we began to notice that too often our mutilated patients were returned to us with the same old disturbing symptoms, and requiring of us the same old fight for relief, until finally it dawned on us that possibly our more progressive brethren really didn't know it all after all. This sort of unpleasant surprise came so often to us and our patients, that very naturally we became presumptious, and would refuse at times to concur in the required mutilation. When, in such cases, we found that more careful study of the individual condition, and more thorough and comprehensive therapy would a great many times enable us to succeed in our efforts, we began to sit up and take notice, and now the tendency is more and more to put ourselves in the attitude of the man from Missouri: we want to be shown; and we want, whenever it is humanly possible, to have a diagnosis furnished us before the abdomen is opened, not after.

That there is much that passes under the name of surgery being done by ill-trained, incompetent men, will not be denied. There will always be room for the young surgeon who, fitted by nature for the work, takes the time and opportunity to prepare himself. There is more good surgery being done than ever before, and there are more good surgeons being educated to do the work. If, however, the surgeon of the future is to hold the high and honored position our leaders have held in the past, there must be some standard of qualification established that shall protect the people against incompetency and dishonesty.

In an address delivered before the American Medical Association in 1905, Dr. Maurice Richardson discusses this subject in such a clear-cut, forceful way that I shall quote him here. He said "The burden of the following remarks is that those only should practice surgery who by education in the laboratory, in the dissecting room, by the bedside and at the operating table, are qualified first, to make reasonably correct deductions from subjective and objective signs; secondly, to give safe and sound advice for or against operations; thirdly, to perform operations skillfully and quickly; and fourthly, to conduct wisely the after treatment."

The task before me is a serious criticism of what is going on in every community, more especially in the smaller centers of population. I do not single out any community, or any man. You remember I stated that I have had this purpose in mind for a number of years, consequently, the idea was given me from an accumulated experience. There is in my mind no doubt whatever that surgery is being practiced by those who are incompetent; by those whose education is imperfect; who lack aptitude; whose environment is such that they can never gain that personal experience which alone will really fit them for what surgery means to-day. Not only are they consequently unprepared to do good operative work, but they are of necessity lacking in that broad experience which alone enables a man to select and sift out the surgical from the non-surgical conditions.

Is it strange that with such men at the head of little three and four bed private hospitals, all over the land, there should be many mistaken diagnoses, and many abdomens opened that should have remained intact? The perfection of technique is the cheapest thing about surgery. Any of us, with a little natural aptitude, and a knowledge of anatomy, can learn to open the volume of a man and remove his appendix, or unsex a woman, in record breaking time. We can have it said of us, as I have heard it said of some, "He can open an abdomen quicker than anyone I ever saw." But is it in the power of any of us to properly and carefully attend to the burdensome duties of a successful physician, and with an occasional little six weeks trip to the great clinics of the East, to become capable, not to understand and follow out the usual technique of an operation, but to properly advise our patients as to whether they do or do not need to be operated on. Is it likely that any of us are so gifted as that?

I want to say, gentlemen, that this unconscious bias towards the use of the knife that so often exists in these near-surgeons, and their inability to accurately determine surgical from non-surgical conditions, is tending to discredit real surgery. The laity is beginning to talk of the remarkable prevalence of appendicitis and allied conditions. It has almost come to the point that abdominal pain must mean abdominal section. I can't believe that it has happened that I have been so unusually lucky in that so small a percentage of my patients require operative procedures. I am sure that it is not aitogether due to mistaken diagnosis, because I do not believe that more of my patients die than those of other men; and I am sure that I have had the same opportunity as other men to find these patients, in that I have been so fortunate as to be a busy man for many years.

Dr. Richardson says—"All surgeons are of course liable to error, not only in diagnosis, but in performance of operations based on diagnosis. Such errors must always be expected and included in the contingencies of the practice of the profession. If, however, serious operations are in the hands of men of special training, or of large experience, such errors will be reduced to a minimum. There is in my mind no doubt whatever that not only is surgery is being doneby these men that is useless—worse than useless. In view of these things, who should perform sugery? How shall the surgeon be fitted for these great duties? As a matter of right and wrong, who shall in the opinion of the medical profession, perform these responsible services, and who shall not? I have no hesitation in saying that the proper fitting of a man for sugical pratice requires a much longer experience as a student and assistant than the most exacting schools demand. A man should serve four, five, or six years, as assistant to an active surgeon, before he can be fully capable of determining, not how, but when, to operate."

We are in danger of running mad along this line. Are drugs to play any part at all in the medicine of the future? There is no doubt that, to a certain extent, the broadening of the field of surgery at the expense of internal medicine has been justifiable. Nevertheless, that there is danger of going too far in our operative furor is shown by the more conservative attitude assumed of late by surgeons of large experience. The removal of the pyloric end of the stomach may be done with impunity so far as the operative mortality is concerned, but it cannot be done with impunity as regards the preservation of the physiological functions of the organ. If a patient with a truncated stomach must ever afterward lead that careful and abstemious life which, in many cases of itself, would have given him the same comfort and security, it is difficult to see how he has been benefitted by surgery. I have even known an intestinal canal to be shortened for the relief of chronic constipation. In the name of our professional fore-fathers, where are we drifting?

With the rapid invasion of these fields by the surgeon it looked for a time as if the internist was doomed to a subordinate place in the scheme of modern medicine. A little more experience with some of the results of this sort of work, however, has served to dissipate this fear.

The time has come for us to give a little more thought to drug therapy, hygiene and dietetics? There was never a time when our use of these useful agents was so accurate as at present. The determination and isolation of active principles, the establishment of definite standards, the growth of animal experimentation, and the extension of clinical observation, are slowly but surely converting the empiricism of the past into the science of the present. We may now accurately control physiological functions, modify metabolic processes, and neutralize or antagonize the products of germ activity. Modern methods of diagnosis make possible not only the determination of signs and symptoms hitherto unrecognized, but likewise enable us to place a proper valuation on them. Take, for instance, the blood pressure apparatus. A few years ago it meant little or nothing to the average physician. Physiologic chemistry, and the microscope, not only tell us certain things about pathologic processes, but enable us to accurately estimate the action and the effect of the remedies we use in our efforts to correct them.

As the representative of that great class of strictly general practitioners—the men who do the hard work of the profession —I want to say that we are of the opinion, and have for a long time been of the opinion, that there is too much surgery; for the three reasons that I have given. In an experience which dates back many years, I have become firmly fixed in the belief that probably twenty-five per cent. of the surgery that is done is unnecessary. Unnecessary because of mistaken diagnosis; unnecessary because of the failure to first resort to the use of ordinary methods of cure; unnecessary in that no appreciable results are obtained.

Let us endeavor, then, in the first place, to protect the people from the ignorant or indifferent medical man. Let us insist upon a more careful and thorough preparation on the part of those who propose to do this sort of work, whether as a side line or specialty. And, finally, let us call a halt to this operative furor which threatens to make near-surgeons of us all.

